# Causes of Trauma in Pregnant Women Referred to Shabih-Khani Maternity Hospital in Kashan

**DOI:** 10.5812/atr.5291

**Published:** 2012-06-01

**Authors:** Elaheh Mesdaghinia, Zahra Sooky, Azam Mesdaghinia

**Affiliations:** 1 Trauma Research Center, Kashan University of Medical Sciences, Kashan, IR Iran; 2 Department of Pharmacology, Faculty of Medicine, Kashan University of Medical Sciences, Kashan, IR Iran

**Keywords:** Domestic Violence, Pregnant Women, Wounds and Injuries

## Abstract

**Background:**

Trauma occurs in 7% of pregnancies and is a major cause of morbidity and mortality in the mother and fetus.

**Objectives:**

The present study was conducted in Kashan in 2009–2010 to evaluate the causes of trauma in pregnancy.

**Patients and Methods:**

This descriptive study analyzed data from 32 pregnant women with trauma who were referred to the maternity hospital from 2009 to 2010. Data included age, gestational age, mother’s occupation, cause of trauma, maternal-fetal complications, gravidity, and parity. The diagnosis of maternal and fetal complications was based on clinical examinations by a gynecologist and results of blood tests, urine analysis tests, and sonography. Data were analyzed as frequency distributions.

**Results:**

the causes of trauma included falling (9 cases (28.1%)), abdominal trauma (8 cases ( 25%)), spousal feud (3 cases (9.4%)), motorcycle accident (2 cases (6.25%)), car accident (2 cases (6.25%)), falling from a motorcycle (2 cases (6.25%)), falling or fainting resulting in head trauma (1 case (3.1%)), pain from crossing over a bump in the car (1 cases (3.1%)), and unspecified causes (4 cases (12.55%)). The causes of traumas occurred between 5 and 40 weeks of gestation. In 17.2% of the cases, trauma occurred prior to 20 weeks of gestation. However, there was no significant relationship between the cause of trauma and maternal age or gestational age. Vaginal bleeding and retroplacental clots were reported in 2 (6.25%) cases and 1 (3.1%) case, respectively.

**Conclusions:**

Nearly half of the women presenting with trauma had experienced spousal feud or domestic violence; therefore, it is necessary to recognize spousal abuse and provide adequate support to traumatized pregnant women.

## 1. Background

Trauma in pregnancy is an important cause of maternal and fetal morbidity and mortality. Trauma occurs in 7% of pregnancies, and at least 5% of fetal deaths are due to the trauma (1). Trauma is among the most frequent causes of non-obstetric maternal and fetal mortality (2). The majority of traumas in pregnancy are mild traumas and carry good maternal and fetal prognoses (3). It has been observed that 84% and 16% of trauma cases include blunt and penetrating traumas, respectively (4). Blunt trauma from car accidents (42%) is the most common cause of trauma in pregnancy (5, 6); however, other studies report that sharp abdominal trauma, such as knife injury, is the most common trauma. Other common causes of trauma include falling from a height and fighting (4). Blunt and penetrating traumas can harm both the uterus and the fetus. If trauma is severe enough to cause an extra-uterine disorder, such as pelvic fracture or retroperitoneal bleeding, it may cause severe side effects, such as fetal brain damage, fetal bone fracture, and placental abruption (5-11). Factors affecting maternal and fetal mortality after trauma include injury to the mother, gestational age at the time of trauma, trauma-induced shock, severe injury to the mother’s abdomen, and coagulopathy (1). The prevalence of trauma in pregnancy in Iran is unknown; however, a recent study reported a 1.1% prevalence of trauma leading to hospitalization (1).

## 2. Objectives

Maternal and fetal complications of trauma in pregnancy were evaluated using patient data in Kashan from 2009 to 2010. Using these data, the present study assessed the status of trauma in Kashan from 2009 to 2010. 

## 3. Patients and Methods 

This descriptive study was conducted on all cases of trauma in pregnant women referred to the maternity hospital of Shabih-khani Maternity flospital, Kashan during 2009 and the first half of 2010. Age, gestational age, job, cause of trauma, maternal and fetal complications, number of pregnancies, and parity were evaluated in the 32 women. Diagnosis of maternal and fetal complications was based on both clinical exams by a gynecologist and results of blood tests, urinary tests, sonography, and patient monitoring. The data were analyzed with SPSS software. The percentages and Mean ± SD were calculated for some variables, and Chi Square tests were used. Data are presented in frequency distribution tables. 

## 4. Results

Of the 32 trauma patients analyzed over the 1.5-year duration of this study, 34.5%, 30.7%, and 6.8% of the cases presented in the autumn, spring, and winter, respectively (Table 1). The women were aged 17–40 years old with a mean and standard deviation of 25.66 ± 6.06 years. Of the subjects, 48.1% were 17–24 years old. The interval between trauma occurrence and admission to the emergency department was 16 ± 5.8 hours. In most cases (91%), the patient presented to the hospital in less than 24 hours. The duration of hospitalization was 0.5 to 6 days with a Mean ± SD of 1.8 ± 1.2 days, 42.1% of the women were hospitalized for 1 and 2 days, respectively. It has been reported that 71%, 13%, 13%, and 3% of the trauma cases were in their first, second, third, and fourth pregnancies, respectively. The majority of trauma resulting from violence was observed in the second pregnancy or greater. In 4 cases, there was no previous abortion. The gestational age at the time of trauma was 5-40 weeks. In 56.3% of the cases, the trauma occurred before 32 weeks’ gestation, and 15.6% occurred before 20 weeks’ gestation. We classified abdominal trauma, spousal feud, and unspecified causes as violent causes. As shown in Table 2, there was a lower incidence of violent trauma in patients with a greater gestational age. There was a higher incidence of violent trauma in older women, although this relationship was not significant. 

**Table 1.** Frequency and Percentage of Distribution of Trauma Causes

Cause of Trauma	No. (%)
Fall	9 (28.1)
Trauma to abdomen	8 (25)
Spousal feud	3 (9.4)
Motorcycle accident	2 (6.25)
Car accident	2 (6.25)
Falling from a motorcycle	2 (6.25)
Falling or fainting resulting in head trauma	1 (3.1)
Pain from crossing a bump in the car	1 (3.1)
Unspecified	4 (12.55)
Total	32 (100)

**Table 2.** Frequency and Percentage of Distribution of Violence by Gestational Age, Maternal Age, and Gravida

Index	Cause of Trauma			
	Violence, No. (%)	Other, No. (%)	Total, No. (%)	Result, No. (%)
Gestational age, week				NS ^a^
1^st^ trimester	1 (100)	0 (0)	1	
2^nd^ trimester	6 (50)	6 (50)	12	
3^rd ^trimester	8 (42.1)	11 (57.9)	19	
Maternal age				NS ^a^
17-24	7 (43.8)	9 (56.3)	16	
25-30	3 (33.3)	6 (66.7)	9	
31-40	5 (71.4)	2 (28.6)	7	
Gravida				NS ^a^
1	9 (39.1)	14 (60.9)	23	
2-4	6 (66.7)	3 (33.3)	9	

^a^Abbreviation: NS, Non-significant

Vaginal bleeding after trauma was detected in 2 cases (6.3%). Placental clot was observed in only one case by sonography. Normal vaginal delivery after trauma was performed in one case at 39 weeks of gestation. Another patient was hospitalized twice after trauma due to the premature rupture of membrane, and the baby had not been delivered by the time the research results were reported. Cesarean delivery was performed on one patient at 33 weeks’ gestation because of fetal distress. 

## 5. Discussion

Our results revealed that 9.4% and 25% of the trauma cases resulted from spousal feud and abdominal trauma (of unspecified cause), respectively. Further, 12.5% of the women did not specify the cause and type of trauma. Together, 46.9% of the cases may be considered a result of domestic violence (Table 1). In the study of Salehi and Mehralian (2009) on women in Shahrekord, Iran, 67.5% of the subjects experienced spousal feud. Of these, 34.5%, 51.7%, and 13.8% were physical, mental, and sexual abuses, respectively (11). The prevalence of spousal feud was reported to to be 60.5% in Khosravi’s study (8). The prevalence of domestic violence among pregnant women referred to the medical university hospital of Iran was as 60.6% (12). Doulatian et al. (2009) reported spousal feud among pregnant women in Gachsaran, Iran (13). Rachana et al. reported that 21% of women in Saudi Arabia experienced physical violence, 78% of which was perpetrated by their spouses (13). There are discrepant reports on the prevalence and type of spousal feuds causing trauma during pregnancy. This may be due to differences in methodology and sampling, cultural differences in reporting trauma, and differences in knowledge of women's rights. Indeed, there are reports demonstrating that the prevalence spousal feud as a cause of trauma may be affected by differences in study methods, sampling techniques, cultural differences in the societies’ attitudes towards women, cultural differences in reporting matrimony experiences, and women’s awareness of their rights. In the present study, the mean age was 25.66 years. In the study by Sperry et al., the mean age of women in the injured group was 24 years, which was significantly higher than the non-injured group (1).

In our previous study in 2007 in Kashan, Iran, we reported a mean age of 26.3 years in the trauma patients, which is similar to that in the present report (9). The similarity in the mean age and the fact that the subjects were young might be attributed to both the prevalence of pregnancies in young women and the high rate of accidents among this age group. In the present study, we did not find a relationship between the age at trauma and the kind of the trauma (presence or absence of domestic violence). However, there was a higher prevalence of violence in older mothers. All women in this study were housekeepers. Khosravi et al. reported that housekeepers were more likely subjected to spousal feuds than employed women (8). Rathora et al. reported similar conclusions (14). In a 2000 UNICEF report, women’s economical dependence was highlighted as a major factor contributing to violence (15). In this study, we did not detect a significant relationship between the gestational age and violence in pregnancy, although there was a higher prevalence of violence during early gestational age. Babapour et al. reported that there was no significant difference in mean gestational age of the fetus between those with or without violent trauma (16). This finding is consistent with those reported by Bodaghabadi in Sabzevar, and by Leung et al. in China (17, 18). However, Neggers et al. reported that, in America, women suffering from severe violence were more likely to have an early delivery compared to those without violence (19). The discrepancy in these results may be associated with the different effect of violence on the pregnancy. Neggers et al. analyzed the relationship between physical violence-induced injury and gestational age; in contrast, our study evaluated the types of traumas regardless of whether or not they were resulted in injury. In the present study, the 2 cases of vaginal bleeding after trauma were associated with physical violence committed by the husband. Bagherzadeh et al. (2008) identified a relationship between physical and mental violence in pregnancy and hospitalization due to vaginal bleeding in the second and third trimesters of pregnancy (20). A correlation between mental violence in pregnancy and vaginal bleeding in the first trimester of pregnancy was also identified by Kearney et al. (21). In the present study, fetal movements decreased because of abdominal trauma in 1 case (3.1%), because of a motorcycle crash in another (3.1%), and because of a placental clot in the third (3.1%). None of these causes was associated with early delivery until the end of the study. Mesdaghinia et al. reported placental abruption in one case (1.66%) (9). Schiff et al. studied 625 pregnant women hospitalized after motorcycle crashes. They reported 2 cases with severe injuries resulting in death; however, there were no significant differences in complications, such as early delivery, placenta abruption, or preterm cesarean section, between those with mild and severe trauma to the abdomen. It seems that mild trauma can contribute to placental placenta abruption or stimulate early delivery by compressing the fetus, resulting in gestational complications. Thus, supporting mothers with mild trauma is important (6). In our study, we identified a higher incidence of violence in mothers in their second pregnancies than in those in their first pregnancy. However, there was no relationship between gravida and violence. Bagherzadeh et al. (2008) reported no statistically significant relationship between the type of violence and number of pregnancies (20). Similarly, Doulatian et al. found no relationship between the number of pregnancies and the presence of abuse. However, Salehi and Mehralian (2009) reported a significantly elevated rate of violence in women in their first pregnancies (11). 

It is important to detect domestic violence and support the vulnerable pregnant women, because approximately half of the women in this study suffered from spousal feuds and/or domestic violence.
